# Lappaol F regulates the cell cycle by activating CDKN1C/p57 in human colorectal cancer cells

**DOI:** 10.1080/13880209.2023.2172048

**Published:** 2023-01-28

**Authors:** Rui-Yi Yang, Jia-Yi Tan, Zhe Liu, Xiao-Ling Shen, Ying-Jie Hu

**Affiliations:** Science and Technology Innovation Center, Guangzhou University of Chinese Medicine, Guangzhou, China

**Keywords:** Cyclin-dependent kinase inhibitor 1C/p57, cyclin-dependent kinase, cyclin

## Abstract

**Context:**

Lappaol F (LAF), a natural lignan from *Arctium lappa* Linné (Asteraceae), inhibits tumor cell growth *in vitro* and *in vivo.* The underlying mechanism involves the suppression of the Yes-associated protein. However, the specific role of LAF in cell cycle regulation remains unknown.

**Objective:**

This study determined the molecular mechanism by which LAF regulates cell cycle progression.

**Materials and methods:**

Various colon cancer cell lines (SW480, HCT15, and HCT116) were treated with LAF (25, 50, and 75 μmol/L) for 48 h. The effects of LAF on cell proliferation and cell cycle were determined using sulforhodamine B and flow cytometry assays. Differentially expressed proteins (DEPs) were identified using quantitative proteomics. Bioinformatic analysis of DEPs was conducted *via* Gene Ontology (GO) and Kyoto Encyclopedia of Genes and Genomes (KEGG) analyses. Expression levels of DEPs in the cell cycle pathway were analyzed using RT-qPCR and western blotting.

**Results:**

LAF suppressed the proliferation of SW480, HCT15, and HCT116 cells (IC_50_ 47.1, 51.4, and 32.8 μmol/L, respectively) and induced cell cycle arrest at the S phase. A total of 6331 proteins were identified and quantified, of which 127 were differentially expressed between the LAF-treated and untreated groups. GO and KEGG enrichment analyses revealed that DEPs mainly participated in the cell cycle. CDKN1C/p57 showed the most significant differential expression, with the highest fold-change (3.155-fold). Knockdown of CDKN1C/p57 attenuated the S phase cell cycle arrest and proliferation inhibition induced by LAF.

**Conclusion:**

LAF exerts antitumor effects *via* S phase arrest by activating CDKN1C/p57 in colorectal cancer cells.

## Introduction

Colorectal cancer (CRC) is the third most common cancer and second leading cause of cancer-related deaths worldwide. Cancer statistics from 185 countries revealed that CRC accounted for 10% of all new cancer cases and 9.4% of all cancer-related deaths in 2020 (Sung et al. 2021). Although the overall survival rate of patients with CRC has improved with advances in treatment strategies, such as chemotherapy, the success of treatment is still impeded by drug resistance and adverse reactions. Hence, medicinal plants are being actively studied to identify new anticancer agents that exhibit high sensitivity with few side effects.

Fructus arctii, the dried ripe fruit of *Arctium lappa* Linné (Asteraceae), is a traditional medicine used for the treatment of cough, inflammation, diabetes, and cancer (Li et al. 2022). Arctigenin, one of the main components of fructus arctii, has been shown to exert anticancer effects in various cell types, including metastatic colorectal cells (Han et al. 2016). Lappaol F (LAF) is another antitumor compound identified from fructus arctii (Sun et al. 2014; Li et al. 2021). We previously reported that LAF significantly suppresses the growth of colon cancer cells *in vitro* and *in vivo*. The underlying mechanism involves the inhibition of the Yes-associated protein (YAP) (Li et al. 2021). YAP is the core effector of the Hippo signaling pathway. As an evolutionarily conserved regulatory network, the key functions of the Hippo signaling pathway include organ growth control, stem cell differentiation, tissue regeneration, and cell cycle regulation (Ehmer and Sage 2016; Lee et al. 2018). Dysregulation of the Hippo pathway leads to hyperactivity of YAP, negatively affecting the cell cycle in many ways, such as by activating the 2 factor and cyclin-dependent kinases (CDKs), suppressing cyclin-dependent kinase inhibitors (CDKNs), stimulating polyploidization, increasing the transcription of DNA synthesis genes, and enhancing DNA repair, ultimately promoting tumor initiation and/or progression (Xiao and Dong 2021). Although LAF has been shown to arrest tumor growth as a YAP inhibitor, its role in cell cycle regulation has not yet been elucidated. Therefore, in this study, the effects of LAF on the cell cycle in colon cancer cells were investigated using quantitative proteomics. We found that LAF induced cell cycle arrest by regulating the cyclin-dependent kinase inhibitor 1 C (CDKN1C/p57), CDKs (CDK1/2/6), and cyclins (cyclin A2 [CCNA2] and cyclin B1 [CCNB1]).

## Materials and methods

### LAF preparation

**Figure 1.**
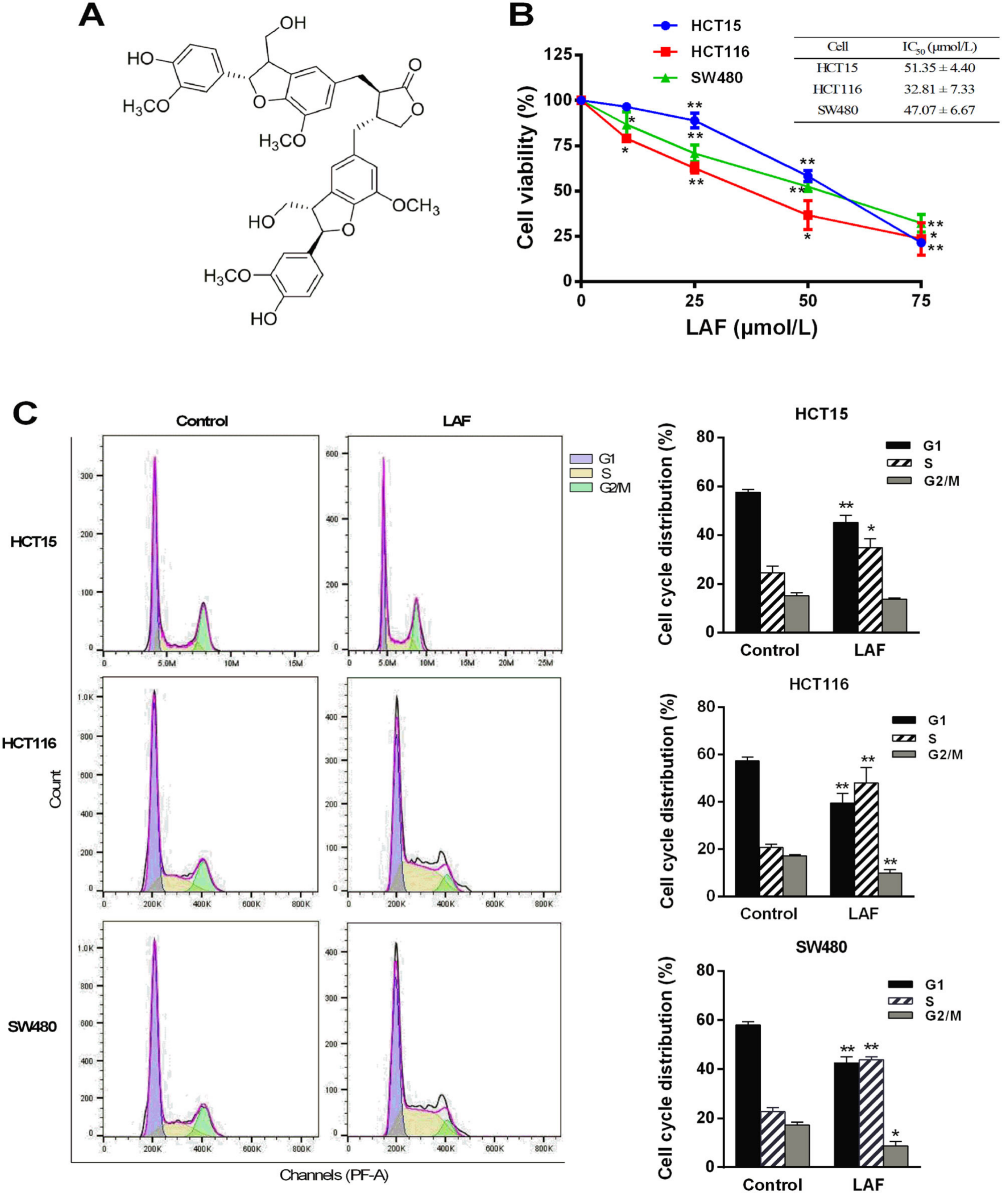
LAF inhibits proliferation and induces the S phase arrest of human colorectal cancer cells. (A) Structure of LAF. (B) Human colon cancer cell lines (HCT15, HCT116, and SW480) were treated with LAF (0, 25, 50 or 75 μmol/L) for 48 h. Cell viability was assessed *via* sulforhodamine B assay. (C) HCT15, HCT116, and SW480 cells were treated with 50 μmol/L LAF for 48 h and then analyzed *via* flow cytometry after propidium iodide staining. All data are expressed as the mean ± standard deviation (SD) (*n* = 6). *p < 0.05, **p < 0.01, significantly different from the control without LAF treatment.

Seeds of *Arctium lappa* were collected from Guangzhou (Guangdong Province, China) in August 2014 and identified by Dr. YJ Hu of the Guangzhou University of Chinese Medicine (Guangzhou, China). A voucher specimen (201408-nbz) was deposited in the Chinese Herbal Drug Discovery Laboratory of the Science and Technology Innovation Center, Guangzhou University of Chinese Medicine. LAF (C_40_H_42_O_12_; Mt = 714; Figure 1(A)) was extracted as previously described (Sun et al. 2014; Li et al. 2021). Its yield and purity were determined to be 0.03 and 98.0%, respectively, *via* high-performance liquid chromatography (HPLC). LAF was dissolved in dimethyl sulfoxide as a stock solution and diluted with the cell growth medium to obtain the working concentration before use.

### Cell culture and proliferation assay

Human colon cancer cell lines (HCT15, HCT116, and SW480) were obtained from the American Type Culture Collection (Manassas, VA, USA) and cultured in the Roswell Park Memorial Institute-1640 medium supplemented with 10% fetal bovine serum (FBS). For proliferation assays, cells were seeded in a 96-well plate (8.0 × 10^3^ cells/well). After treatment with LAF, sulforhodamine B (Sigma-Aldrich, St. Louis, MO, USA) assay was performed to assess the cell proliferation.

### Cell cycle analysis via flow cytometry

HCT15, HCT116, and SW480 cells were seeded in a 6-well plate (3.75 × 10^5^ cells/well) and treated with 50 μmol/L LAF for 48 h. Cells were trypsinized, collected, washed with cold phosphate buffered saline (PBS), and fixed overnight in absolute ethanol at −20 °C. Subsequently, the cells were washed, resuspended in PBS, and stained with a DNA staining solution containing propidium iodide (PI) and RNAase (Multi Sciences, Hangzhou, China) for 30 min in the dark at 25 °C. Cellular DNA content was determined *via* flow cytometry using a Cyto FLEX flow cytometer (Beckman Coulter, Brea, CA, USA) and analyzed using the FlowJo v10.7.1 software (Becton, Dickinson and Company, Ashland, OR, USA).

### Quantitative analysis of the global proteome

SW480 cells were cultured in 25-cm^2^ culture bottles and treated with 50 μmol/L LAF for 24 h. Protein was extracted, purified, digested with trypsin, and labeled using a 6-plex tandem mass tags (TMT) kit (ThermoFisher Scientific, Rockford, IL, USA). Labeled peptides were separated into fractions *via* high-pH reverse-phase HPLC and then analyzed *via* liquid chromatography-tandem mass spectrometry. These tests were performed by PTM Biolabs (Hangzhou, China). The mass spectrometry proteomics data of this study have been deposited in the ProteomeXchange Consortium *via* the PRIDE partner repository with the dataset identifier PXD017872 (http://www.ebi.ac.uk/pride/archive/projects/PXD017872).

### Reverse transcription-quantitative polymerase chain reaction (RT-qPCR)

Total RNA was isolated from cells using the TRIzol reagent, and residual DNA was digested with RNase-free DNase I. First-strand complementary DNA (cDNA) was synthesized using the PrimeScripr RT reagent kit (TaKaRa Bio, Dalian, China), according to the manufacturer’s protocol. Quantitative PCR was performed using the TB Green Premix Ex Taq II kit (TaKaRa Bio) with the 7500 real-time PCR system (Applied Biosystems, Foster City, CA, USA). All primer sequences used for PCR analysis in this study are listed in Table 1. Glyceraldehyde-3-phosphate dehydrogenase was used as an internal reference.

**Table 1.** Primers for quantitative PCR analysis.

Genes	Primer sequences (5′-3′)
CDKN1C/p57	F: AACGCCGAGGACCAGAACC
	R: GCGAAGAAATCTGCACCGTCT
CDK1	F: GGTACCTATGCTGGTGCCAGTGCC
	R: CATCAGAGAAAGCCTGACACAGGT
CDK2	F: CCAGGAGTTACTTCTATGCCTGA
	R: TTCATCCAGGGGAGGTACAAC
CDK6	F: GCTGACCAGCAGTACGAATG
	R: GCACACATCAAACAACCTGACC
CCNA2	F: CGCTGGCGGTACTGAAGTC
	R: GAGGAACGGTGACATGCTCAT
CCNB1	F: AATAAGGCGAAGATCAACATGGC
	R: TTTGTTACCAATGTCCCCAAGAG
TTK	F: TCATGCCCATTTGGAAGAGTC
	R: CCACTTGGTTTAGATCCAGGC
BUB1B	F: AAATGACCCTCTGGATGTTTGG
	R: GCATAAACGCCCTAATTTAAGCC
CHEK1	F: ATATGAAGCGTGCCGTAGACT
	R: TGCCTATGTCTGGCTCTATTCTG
BUB1	F: AGCCCAGACAGTAACAGACTC
	R: GTTGGCAACCTTATGTGTTTCAC
CDC20	F: GCACAGTTCGCGTTCGAGA
	R: CTGGATTTGCCAGGAGTTCGG
CDC25C	F: ATGACAATGGAAACTTGGTGGAC
	R: GGAGCGATATAGGCCACTTCTG
PLK1	F: CACCAGCACGTCGTAGGATTC
	R: CCGTAGGTAGTATCGGGCCTC
GAPDH	F: GGAGCGAGATCCCTCCAAAAT
	R: GGCTGTTGTCATACTTCTCATGG

BUB1: BUB1 mitotic checkpoint serine/threonine kinase; BUB1B: BUB1 mitotic checkpoint serine/threonine kinase B; CDC20: cell division cycle 20; CDC25C: cell division cycle 25 C; CCNA2: cyclin A2; CCNB1: cyclin B1; CDK1: cyclin-dependent kinase 1; CDK2: cyclin- dependent kinase 2; CDK6: cyclin-dependent kinase 6; CDKN1C/p57: cyclin-dependent kinase inhibitor 1 C; CHEK1: checkpoint kinase 1; GAPDH: glyceraldehyde-3-phosphate dehydrogenase; PLK1: polo like kinase 1; TTK: TTK protein kinase.

### Western blotting

Total protein was extracted from cells using the radioimmunoprecipitation assay lysis buffer containing phenylmethylsulfonyl fluoride at a final concentration of 1 mmol/L (Beyotime Co, Shanghai, China). Western blotting was performed using antibodies against CDKN1C/p57 (Cell Signaling Technology [CST]; #2557), CDK1 (Abcam; ab133327), CDK2 (CST; #2546), CDK6 (CST; #3136), CCNA2 (CST; #4656), and CCNB1 (CST; #12231), as previously described (Song et al., 2019). β-Actin (Abcam; ab8226) was used as an endogenous reference.

### CDKN1C/pP57 knockdown in SW480 cells

To knockdown CDKN1C/p57 expression, a lentivirus expressing a small interfering RNA (siRNA) specific for CDKN1C/p57 gene (NM_001122631) was prepared by GeneChem (Shanghai, China). The day before infection, SW480 cells were seeded in a 24-well plate and cultured until they reached 20–30% confluency. The virus was diluted in a serum-free medium (multiplicity of infection = 50) and incubated with the cells for 16 h. After removing the virus dilution, the cells were cultured in a medium containing 10% FBS for another 72 h. Then, fresh medium containing puromycin (2 μg/mL) was replaced every three days to screen for cells stably expressing the CDKN1C/p57-siRNA. Protein expression was determined *via* western blotting. Negative control siRNA lentivirus was used as the control.

### Statistical analysis

Results are expressed as the mean ± standard deviation. Comparisons between groups were analyzed *via* the Student’s *t-*test or one-way analysis of variance with *post hoc* Tukey’s test using SPSS software (version 24.0; SPSS, Inc., Chicago, IL, USA). *p*** **<** **0.05 was considered to be statistically significant.

## Results

### LAF induces S phase arrest and inhibits the proliferation of human CRC cells

LAF decreased the viability of human colon cancer cells. Half-maximal inhibitory concentration (IC_50_) values of LAF in HCT15, HCT116, and SW480 cells were 51.4, 32.8, and 47.1 μmol/L, respectively, at 48 h (Figure 1(B)). To determine whether LAF regulated the cell cycle, cells treated with or without LAF were stained with PI and subjected to flow cytometric analysis. The results showed that LAF significantly increased the proportion of cells in the S phase, while decreasing the proportion of cells in the G2/M phase in HCT15, HCT116, and SW480 cells. After treatment with 50 μmol/L LAF for 48 h, the average ratio of the S phase increased by 1.4-, 2.3-, and 2.0-times in HCT15, HCT116, and SW480 cells, respectively. These results suggest that LAF induces cell cycle arrest in the S phase (Figure 1(C)).

### Bioinformatic analysis of proteomic data

**Figure 2.**
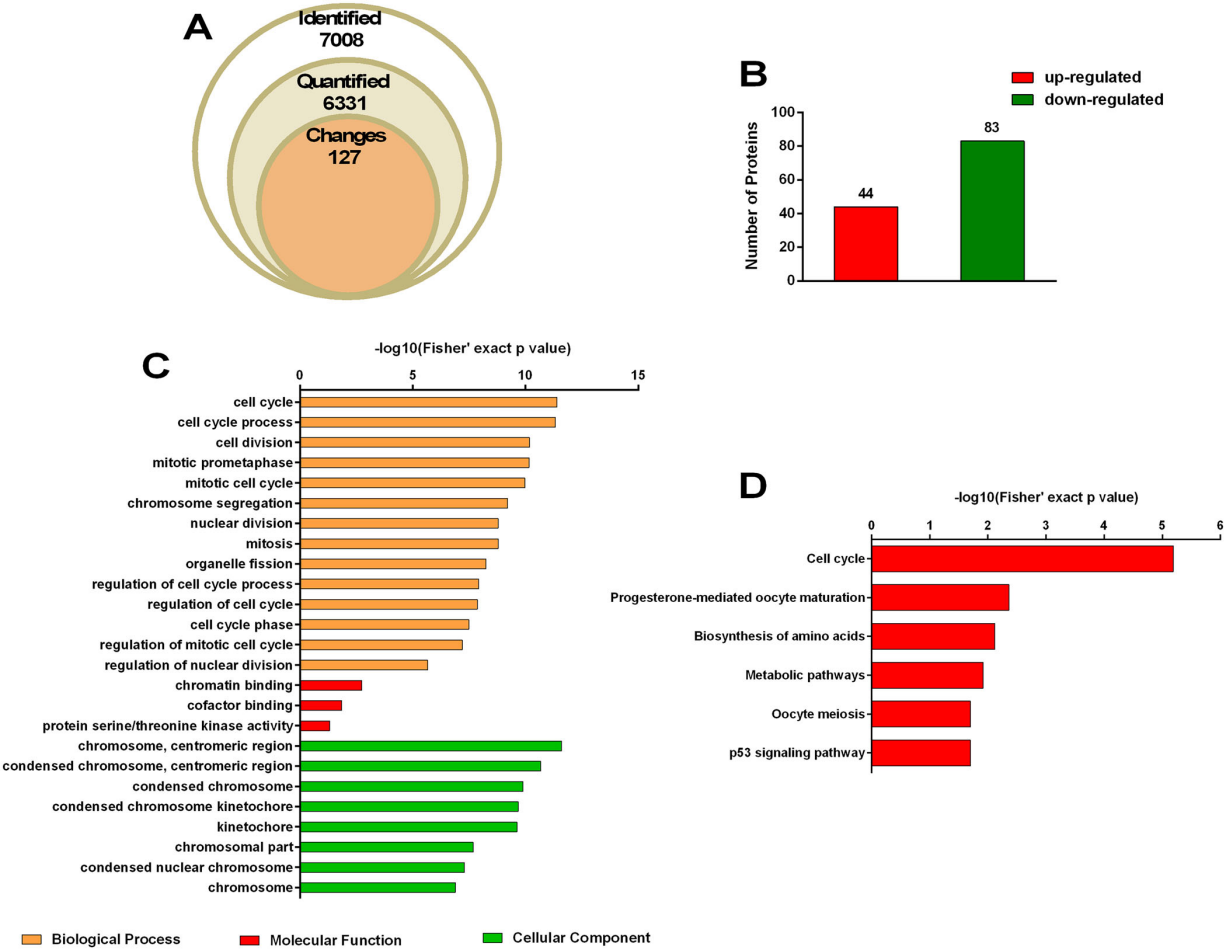
Proteome profiling. SW480 cells were treated with 50 μmol/L LAF for 48 h. Proteins were quantified *via* tandem mass tag (TMT) labeling and liquid chromatography-tandem mass spectrometry (LC-MS/MS) analysis. Fold change ≥ 1.5 and *p* value < 0.05 were considered to indicate differential expression. There were three biological replicates in each group. (A) Quantitative overview. (B) Differentially expressed proteins (DEPs). (C) Gene Ontology (GO) enrichment analysis of DEPs. (D) Kyoto Encyclopedia of Genes and Genomes (KEGG) pathway enrichment analysis of DEPs.

We identified a total of 7008 proteins in our proteomic analysis, of which 6331 were quantified (Figure 2(A)). Compared to the untreated group, fold change ≥1.5 and *p*-value < 0.05 were considered to indicate differential expression. A total of 127 differentially expressed proteins (DEPs), including 44 upregulated and 83 downregulated proteins, were analyzed for functional enrichment (Figure 2(B)). Fourteen relevant biological processes including the cell cycle (*p*** **=** **4.044 × 10^−12^, 45 associated proteins), cell cycle process (*p*** **=** **4.77 × 10^−12^, 41 associated proteins), regulation of cell cycle process (*p*** **=** **1.197 × 10^−8^, 21 associated proteins), regulation of cell cycle (*p*** **=** **1.395 × 10^−8^, 27 associated proteins), and cell cycle phase (*p*** **=** **3.285 × 10^−11^, 21 associated proteins) were identified *via* Gene Ontology (GO) enrichment analysis (Figure 2(C)). Moreover, six pathways, namely the cell cycle (*p*** **=** **6.463 × 10^−6^, 10 associated proteins), progesterone-mediated oocyte maturation (*p*** **=** **4.409 × 10^−3^, 5 associated proteins), biosynthesis of amino acids (*p*** **=** **7.587 × 10^−3^, 5 associated proteins), metabolic pathways (*p*** **=** **1.215 × 10^−2^, 18 associated proteins), oocyte meiosis (*p*** **=** **1.974 × 10^−2^, 5 associated proteins), and p53 signaling pathway (*p*** **=** **2.011 × 10^−2^, 4 associated proteins) were found to be highly enriched *via* Kyoto Encyclopedia of Genes and Genomes (KEGG) analysis (Figure 2(D)).

### DEPs are involved in the cell cycle pathway

Enrichment analysis revealed that, 10 DEPs namely CDKN1C/p57, CCNA2, CCNB1, TTK protein kinase (TTK), BUB1 mitotic checkpoint serine/threonine kinase B (BUB1B), checkpoint kinase 1 (CHEK1), BUB1 mitotic checkpoint serine/threonine kinase (BUB1), cell division cycle 20 (CDC20), cell division cycle 25 C (CDC25C), and polo like kinase 1 (PLK1), were enriched in the cell cycle pathway, among which CDKN1C/p57 was upregulated, while all others were downregulated (Table 2). To verify the reliability of the proteomic data and further understand the correlation between proteins and their encoding genes, the mRNA levels of these 10 proteins were determined *via* RT-qPCR. The transcripts of genes encoding three cyclin-dependent kinases (CDK1, CDK2, and CDK6) with a change between 1.2- and 1.5-fold were quantified simultaneously. The results showed that the expression patterns (upregulation or downregulation) of the 12 genes (except CHEK1) at the mRNA level were consistent with those of the protein in proteomic analysis (Table 2). Expression of CDKN1C/p57, the most significant DEP with the highest fold-change (3.155, *p*** **=** **0.000468) in proteomic analysis, was also significantly elevated in RT-qPCR analysis.

**Table 2.** Differentially expressed proteins in the cell cycle pathway.

Protein accession	Protein name	Fold-change in proteomic analysis	Fold-change in RT-qPCR
P49918	CDKN1C/p57	3.155	2.360
P33981	TTK	0.637	0.934
O60566	BUB1B	0.634	0.425
O14757	CHEK1	0.633	1.800
O43683	BUB1	0.618	0.616
Q12834	CDC20	0.563	0.347
P30307	CDC25C	0.545	0.531
P53350	PLK1	0.518	0.263
P14635	CCNB1	0.498	0.523
P20248	CCNA2	0.438	0.620
P06493	CDK1	0.837	0.735
P24941	CDK2	0.811	0.667
Q00534	CDK6	0.683	0.594

Fold-change represents the LAF-treated group vs. untreated group.

### Effect of CDKN1C/p57 on LAF-induced inhibition of human CRC proliferation

**Figure 3.**
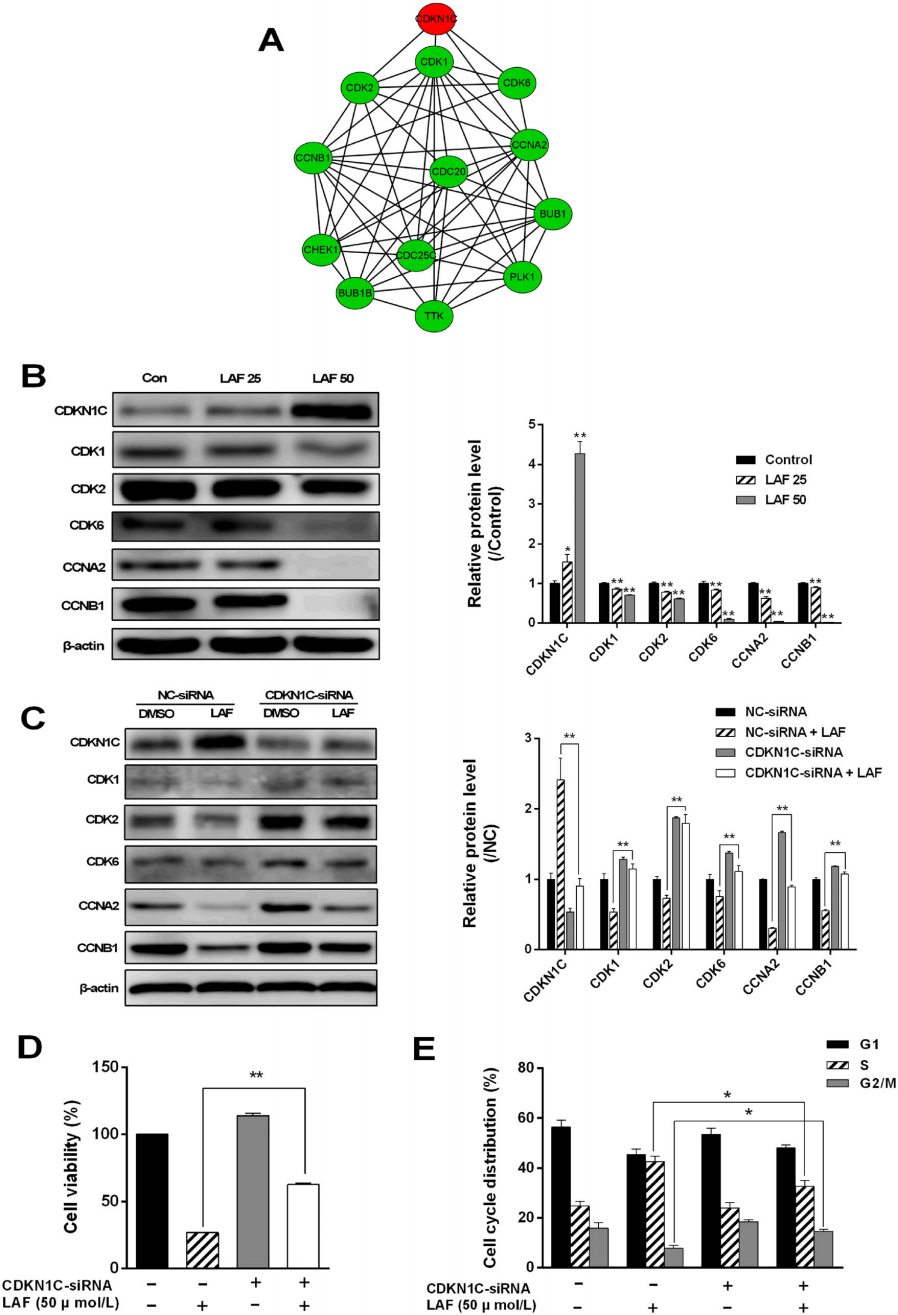
LAF inhibits colorectal cancer cell proliferation by upregulating the expression of cyclin-dependent kinase inhibitor 1 C (CDKN1C/p57). (A) Protein–protein interaction (PPI) network of differentially expressed proteins in cell cycle pathway (red indicates upregulation, green indicates downregulation). (B) Expression levels of CDKN1C/p57, cyclin-dependent protein kinases (CDKs), and cyclins in SW480 cells after treatment with 25 or 50 μmol/L LAF for 48 h determined *via* western blotting (*p < 0.05, **p < 0.01, significantly different from the control without LAF treatment; *n* = 3). (C) Role of CDKN1C/p57 in LAF-induced inhibition of CDK and cyclin expression. (D) Role of CDKN1C/p57 in LAF-induced cell proliferation inhibition. (E) Role of CDKN1C/p57 in LAF-induced cell cycle arrest. Cells infected with lentiviruses containing CDKN1C/p57-small interfering RNA (siRNA) or negative control (NC)-siRNA were treated with or without LAF (50 μmol/L) for 48 h. Protein levels, cell viability, and cell cycle distributions were assessed *via* western blotting, sulforhodamine B assay, and flow cytometry after propidium iodide staining, respectively (*p < 0.05, **p < 0.01, significantly different between the NC-siRNA- and CDKN1C/p57-siRNA-transfected cells). Western blotting and cell cycle analysis, *n* = 3; cell viability, *n* = 6.

A protein–protein interaction network of DEPs in the cell cycle pathway was constructed using the STRING database (https://cn.string-db.org, interaction score ≥ 0.9), and further visualized using Cytoscape software (v3.8.2). As shown in Figure 3(A), CDKN1C/p57 directly interacted with CDK1, CDK2, CDK6, and CCNA2 and indirectly interacted with CCNB1, TTK, BUB1B, CHEK1, BUB1, CDC20, CDC25C, and PLK. Western blotting results showed that CDKN1C/p57 expression was sharply increased after LAF treatment. However, the expression levels of CDK1, CDK2, CDK6, CCNA2, and CCNB1 showed the opposite pattern (Figure 3(B)). To further explore the role of CDKN1C/p57 in the anti-proliferative effect of LAF, CDKN1C/p57 was knocked down using siRNA in SW480 cells. Compared to the negative control-siRNA-transfected group, CDKN1C/p57-siRNA-transfected cells displayed smaller increase in the expression of CDKN1C/p57 after LAF treatment. Moreover, inhibition of the expression of CDK1, CDK2, CDK6, CCNA2, and CCNB induced by LAF after CDKN1C/p57 silencing was lower than that observed in the negative control group (Figure 3(C)). In addition, knockdown of CDKN1C/p57 significantly reduced S-phase arrest and restored the cell viability (Figure 3(D,E)). These findings suggest that CDKN1C/p57 activation is involved in LAF-induced cell cycle arrest and inhibition of cell proliferation.

## Discussion

We previously reported that LAF suppresses cancer cell proliferation by inhibiting YAP (Li et al. 2021). YAP, a transcriptional activator, promotes cell proliferation by activating the expression of cell cycle genes (Ehmer and Sage 2016). In this study, we showed that LAF causes cell cycle arrest in human CRC cells. Moreover, bioinformatic analysis revealed that LAF-induced DEPs were mainly associated with the cell cycle.

The normal cell cycle sequentially goes through G1, S, G2, and M phases for cell proliferation. The transition between the cell cycle phases of eukaryotic cells is governed by the ordered activation of a set of cyclin–CDK complexes, and CDKNs inhibit the activities of these complexes at appropriate check points. Ultra-powerful ‘engines’ (such as cyclins and CDKs) and defective ‘brakes’ (such as CDKNs) lead to uncontrolled cell cycle and excessive cell proliferation (Lim and Kaldis 2013). Therefore, cell cycle dysregulation is a common process in the development of cancer (Peng et al. 2021). Known to date, CDKNs are divided into two distinct families, INK4 and CIP/KIP. Members of the INK4 family (CDKN2A/p16^INK4a^, CDKN2B/p15^INK4b^, CDKN2C/p18 ^INK4c^, and CDKN2D/p19 ^INK4d^) specifically inhibit the activities of CDK4 and CDK6, whereas CIP/KIP members (CDKN1A/p21^CIP1^, CDKN1B/p27^KIP1^, CDKN1C/p57^KIP2^, and CDKN3^CIP2^) control a broad spectrum of cyclin–CDK complexes. In this study, CDKN1C/p57 was the most significantly upregulated among all 127 LAF-induced DEPs. CDKN1C/p57, which is highly homologous to CDKN1B/p27, has also been identified as a tumor suppressor (Lee et al. 1995; Matsuoka et al. 1995). Low expression of CDKN1C/p57 has been observed in many types of tumors and is significantly correlated with poor prognosis and aggressive form of the disease (Pateras et al. 2009; Borriello et al. 2011; Lai et al. 2021). During colorectal carcinogenesis, CDKN1C/p57 downregulation is associated with the transition from adenoma to carcinoma and large size of the tumor (Noura et al. 2001; Li et al. 2003). The association between cancer and CDKN1C/p57 expression suggests that increase in CDKN1C/p57 levels may be advantageous for cancer therapy. Similar to CDKN1A/p21 and CDKN1B/p27, CDKN1C/p57 inhibits a series of cyclin–CDK complexes, including CCNE–CDK2, CCNA–CDK2, CCNE–CDK3, CCND1–CDK4, CCND2–CDK4, and to a lesser extent, CCNB–CDK1 and CCND2–CDK6 (Lee et al. 1995; Matsuoka et al. 1995; Reynaud et al. 2000). CCNA–CDK2 has previously been identified as an imperative kinase for DNA replication and S-phase progression (Fotedar et al. 1996; Morgan 2008). In addition, CCNA–CDK2 regulates the timing of CCNB–CDK1 activation during the late S/G2 phase and mediates the S phase exit and G2 phase transition of cells (Oakes et al. 2014). Downregulation of CCNA2 and CDK2 induces the cell cycle at S phase (Cheng et al. 2020). Consistent with the upregulation of CDKN1C/p57 by LAF, the expression of CCNA2, CDK2, CCNB1, and CDK1 was subsequently inhibited, leading to S-phase arrest in SW480 CRC cells.

Upregulation of CDKN1C/p57 mRNA levels was also observed in RT-qPCR, suggesting that LAF activates CDKN1C/p57 at the transcriptional level. To date, only a few studies have investigated CDKN1C/p57 and its regulatory mechanism remains unknown. Unlike CDKN1A/p21 and CDKN1B/p27, the transcription of CDKN1C/p57 is not regulated by p53 (Matsuoka et al. 1995). CDKN1C/p57 is highly expressed in YAP-ablated developing lens epithelial cells (Song et al. 2014). Moreover, YAP silencing or depletion increases the expression levels of CDKN1A/p21 and CDKN1B/p27 in esophageal squamous cell carcinoma and thyroid papillary carcinoma cells (Muramatsu et al. 2011; Liu et al. 2017). Interestingly, YAP has recently been found to function as a transcriptional repressor in addition to its known function as a transcriptional activator. YAP binds to the promoter of CDKN1B/p27 and recruits the multifunctional transcription factor YY1 and polycomb repressive complex 2 subunit enhancer of zeste homologue 2 (EZH2), thereby mediating the transcriptional repression of CDKN1B/p27 (Hoxha et al. 2020). Additionally, CDKN1C/p57 has been demonstrated to be a direct target of EZH2 and is strongly activated by EZH2 single or EZH1/2 dual inhibition (Yang et al. 2009; Li et al. 2019; Li et al. 2020). We previously found that LAF promotes CDKN1A/p21 and CDKN1B/p27 transcription in a p53-independent manner (Sun et al. 2014). Together with the results of our previous study, this study revealed that LAF, as a YAP inhibitor, activated the transcription and expression of CDKN1A/p21, CDKN1B/p27, and CDKN1C/p57, resulting in cell cycle arrest and inhibition of cancer cell proliferation. However, whether LAF activates the three CIP/KIP family members *via* the same YAP-mediated mechanism (such as the EZH1/2 pathway) requires further investigation.

## Conclusion

Our previous study revealed that LAF, an anticancer agent, exerts inhibitory action on YAP. In this study, the effect of LAF on colon cancer cell proliferation was investigated using a proteomic profiling method. In total, 127 DEPs were identified. GO and KEGG enrichment analyses revealed the important roles of LAF in the cell cycle. We found that LAF induced cell cycle arrest by upregulating CDKN1C/p57 expression. Based on the transcriptional promotion effects of LAF on CDKN1A/p21 and CDKN1B/p27 in our previous study, we speculated that LAF may activate CDKN expression *via* a YAP-mediated mechanism, inducing cell cycle arrest and inhibit the proliferation of cancer cells. Our results provide new insights into the antitumor molecular mechanisms of LAF.
